# Rethinking integrated service delivery for malaria

**DOI:** 10.1371/journal.pgph.0000462

**Published:** 2022-06-01

**Authors:** Evelyn K. Ansah, Corrina Moucheraud, Linda Arogundade, Gabriel W. Rangel

**Affiliations:** 1 Centre for Malaria Research, Institute of Health Research, University of Health and Allied Sciences, Ho, Ghana; 2 Department of Health Policy and Management, Fielding School of Public Health, University of California Los Angeles, Los Angeles, California, United States of America; 3 UCLA Center for Health Policy Research, University of California Los Angeles, Los Angeles, California, United States of America; 4 Harvard Kennedy School, Cambridge, Massachusetts, United States of America; 5 Department of Biochemistry and Molecular Biology, Pennsylvania State University, University Park, Pennsylvania, United States of America; UCSI University Kuala Lumpur Campus: UCSI University, MALAYSIA

## Abstract

Despite worldwide efforts and much progress toward malaria control, declines in malaria morbidity and mortality have hit a plateau. While many nations achieved significant malaria suppression or even elimination, success has been uneven, and other nations have made little headway—or even lost ground in this battle. These alarming trends threaten to derail the attainment of global targets for malaria control. Among the challenges impeding success in malaria reduction, many strategies center malaria as a set of technical problems in commodity development and delivery. Yet, this narrow perspective overlooks the importance of strong health systems and robust healthcare delivery. This paper argues that strategies that move the needle on health services and behaviors offer a significant opportunity to achieve malaria control through a comprehensive approach that integrates malaria with broader health services efforts. Indeed, malaria may serve as the thread that weaves integrated service delivery into a path forward for universal health coverage. Using key themes identified by the "Rethinking Malaria in the Context of COVID-19" effort through engagement with key stakeholders, we provide recommendations for pursuing integrated service delivery that can advance malaria control via strengthening health systems, increasing visibility and use of high-quality data at all levels, centering issues of equity, promoting research and innovation for new tools, expanding knowledge on effective implementation strategies for interventions, making the case for investing in malaria among stakeholders, and engaging impacted communities and nations.

## Introduction

Malaria continues to cause significant morbidity and mortality worldwide, but it is preventable and treatable [1]. Over the period 2001 to 2013, a substantial scale-up of malaria interventions globally contributed to a 30% decline in malaria incidence and a 47% decline in malaria mortality, averting an estimated 4.3 million deaths over this period [2].

Following this era of progress and hope, the World Health Assembly adopted the Global Technical Strategy for Malaria 2016–2030 in May 2015, which provides a comprehensive framework to guide countries in their efforts to accelerate progress toward eliminating malaria [2]. World Health Organization (WHO) member states that adopted the strategy endorsed a bold vision of a malaria-free world, with an ambitious target of reducing global malaria incidence and mortality by 90% by 2030.

**Fig 1:**
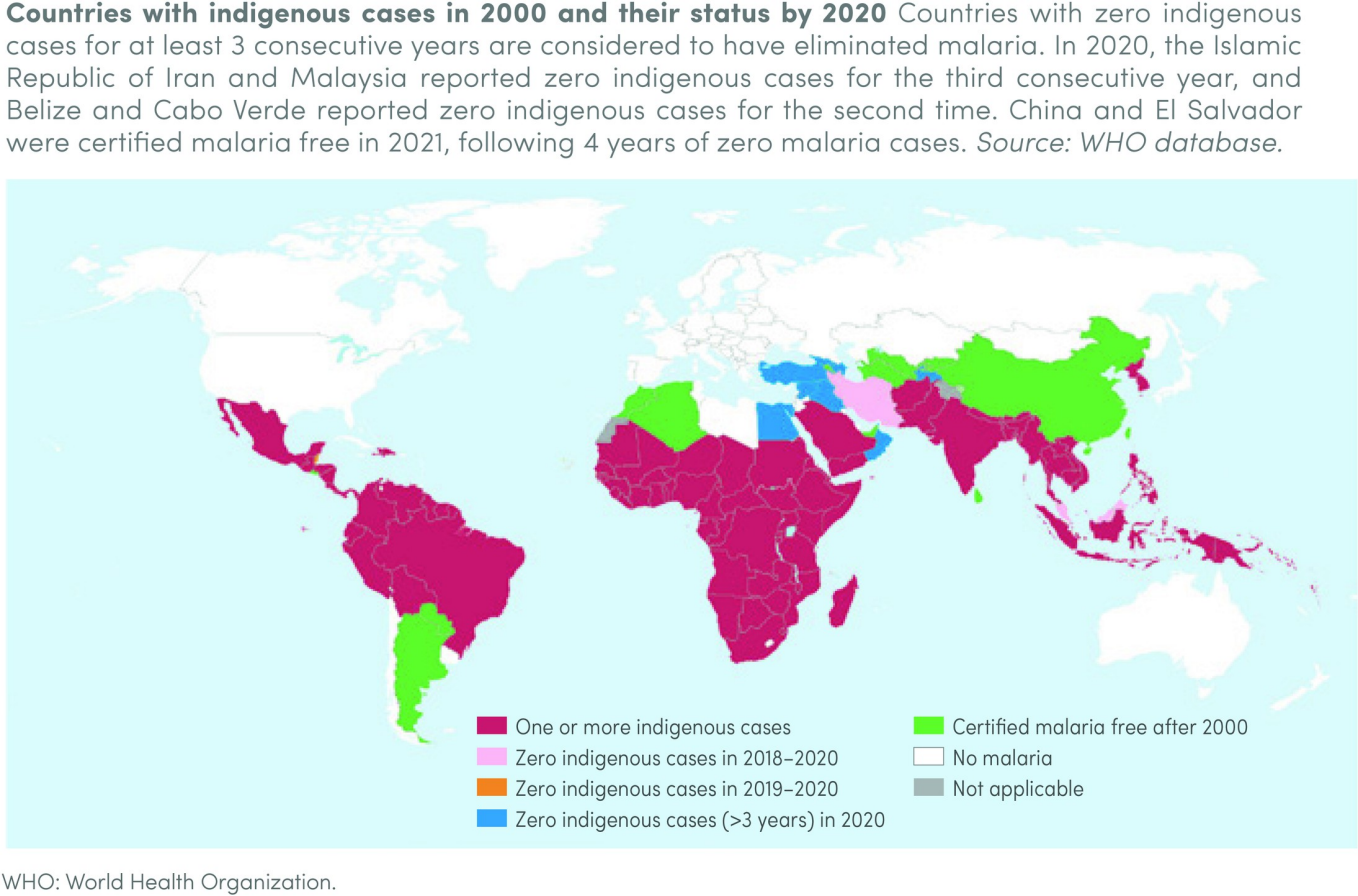
The world’s malaria burden in 2019 (Adapted from the world malaria report 2020).

However, over the first several years of Strategy implementation, progress against malaria mortality and morbidity has slowed, stalled, or reversed in many moderate- and high-transmission countries [3]. Globally from 2015 to 2019, malaria case incidence only declined by an estimated 2%, and the malaria mortality rate declined only marginally, from 12 to 10 deaths per 100,000 population [3]. Further, progress has been heterogeneous across countries and regions (Fig 1). While some countries have made considerable progress, others, particularly in sub-Saharan Africa, have not significantly reduced their malaria burden [3]. Disparate burden in bordering areas can lead to the reintroduction of the disease into countries that have come close to eliminating malaria, further hindering progress.

## The COVID-19 pandemic: Presenting additional challenges and new opportunities

The ongoing COVID-19 pandemic has further threatened the bold ambition of the Global Technical Strategy for Malaria. The health systems of many countries that were already struggling to grapple with malaria, other infectious diseases, and the growing threat of non-communicable diseases were further stressed by COVID-19. Human and other resources originally focused on malaria were diverted to deal with the urgent global public health emergency [4]. There were an estimated 14 million more malaria cases and 47,000 more deaths in 2020 compared to 2019 due to disruptions to services during the pandemic [1].

Yet the COVID-19 pandemic has also re-invigorated the infectious disease agenda. It has underscored the importance of enhancing global collaboration and effective partnerships among all sectors and stakeholders—not only in facing the immense challenges posed by the pandemic but also to ‘build back better’ [5].

### Re-thinking malaria through integrated service delivery

Decades of scientific innovation have generated effective technologies that can be deployed to fight malaria, from prevention to diagnosis to treatment [6]. This armamentarium may predispose some to approach malaria control as a set of technical problems: how can more and better tools be acquired, and how should these tools reach the maximum number of people, including the “unreached?” Although tools are a major challenge, focusing on this alone may be a limited—and limiting—perspective [7, 8]. Tools are critical to the control of infectious diseases, but tools alone cannot solve the whole problem—a lesson reinforced during the COVID-19 pandemic [9]. It similarly may be reductionist to conceptualize the fight against malaria as being merely about product delivery, although the temptation to do so may be exacerbated by some global donors’ tendency to monitor and reward based on commodities [10–12] as it is easier and more immediate to count program outputs (e.g., product distribution, uptake, or coverage) than behavior (e.g., usage or adherence) or health outcomes.

But there are dangers to taking such a technical, commodity-driven approach that decontextualizes malaria control efforts and deemphasizes the role of services and systems. Divorcing malaria products from the systems and services that administer them, as well as their corresponding policies and programs, overlooks important areas that need to be strengthened. Additionally, malaria exists in the context of countless additional health, resource, and political priorities on national, regional, and global levels. Overemphasis on malaria-specific technology development by malaria-focused funders and implementers may limit the malaria community’s engagement in broader dialogues, such as health system strengthening, climate change, and social determinants of health. This takes particular resonance in the context of the global movement toward Universal Health Coverage (UHC). This challenge is not new nor unique to malaria control—in fact, other public health interventions, from immunizations to HIV/AIDS programs—have similarly witnessed how a commodity-driven approach may not be sufficient for achieving actual change. (How the malaria community might learn from these efforts is an area highly worthy of future exploration.)

The WHO recently approved the RTS,S/AS01 malaria vaccine, known as Mosquirix, for widespread use among children in sub-Saharan Africa and other regions with moderate to high *P*. *falciparum* malaria transmission. Whilst this is welcome news to the malaria community as an addition to the toolbox for malaria control, its delivery through another program, the Expanded Program on Immunization (EPI), has the potential to unplug the bottlenecks in acceleration towards malaria elimination. The vaccine, which is meant to be complementary to existing tools for malaria control, is to be delivered through the Expanded Programme of Immunization (EPI), which offers a great opportunity for RTS,S/AS01 to help serve as an example of a malaria intervention that is administered in an integrated fashion outside of the traditional malaria ecosystem through the EPI programme. Though more integrated at the primary care level, these two programs are still not fully integrated, becoming more vertical at higher levels of service delivery, including at the National level.

In practical terms, many malaria services, especially treatment, are often integrated [13]; mothers do not bring their febrile babies to malaria-specific nurses, and drug shops do not sell only antimalarial medications. However, other malaria control activities are less well-integrated (such as mass drug administration), and many high-burden countries lack a systematic approach to integrating the upstream “inputs”—including financing, training, mentorship and supervision, and monitoring and evaluation—to facilitate effective delivery of multiple services downstream [14, 15].

**Fig 2:**
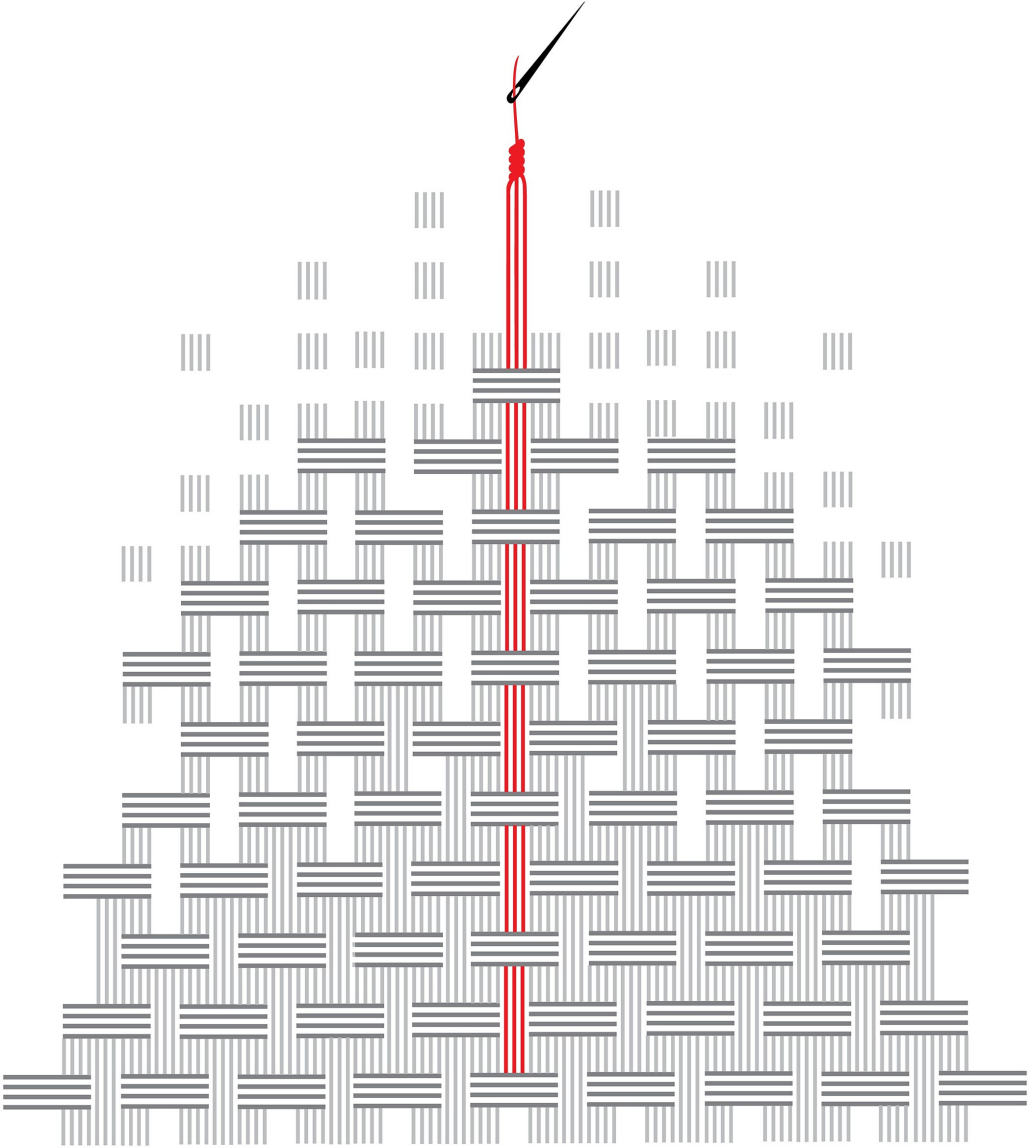
Malaria as the thread around which service integration can be woven towards Universal Health Coverage.

This paper examines the reasons for barriers, and steps needed to move toward integrated service delivery for malaria. Particularly in the context of UHC, there are many unresolved questions about how to expand affordable, high-quality, and cost-effective malaria services while maintaining a focus on reducing the malaria burden and working toward eventual elimination. Although focused programming may be efficient, it does not build robust and resilient health systems capable of providing comprehensive care or weathering unexpected crises [16]. There is an urgent need to consider how malaria efforts could and should be integrated with other services. While there is no one “path” to UHC, integrating services can be the first step. While malaria treatment and diagnosis are often combined with managing other cases at the primary care level, opportunities must be sought to synergize some prevention interventions with other services. Integrating malaria interventions and evaluation efforts with different priorities, especially general health system strengthening, has proven both necessary and impactful [17]. We argue that malaria can be the “thread” that leads the way in integrating services and moving toward UHC (Fig 2).

## Rethinking malaria in the context of COVID-19: Six key themes

“Rethinking Malaria in the Context of COVID-19” was undertaken as a multi-month global engagement process, engaging stakeholders across sectors and disciplines to reflect on malaria progress and where improvement is needed. Service integration was identified as a key area from the outset. We embarked on global dialogue and informal interviews to gather perspectives on this topic, including experiences, challenges, and areas for opportunity. These stakeholders were from the public sector, private sector, multilateral and bilateral agencies, donors, academic institutions, think tanks, and non-governmental implementing organizations; and represented diverse geographic locations, contexts, and backgrounds, both from within and outside the malaria community. This provided a forum to capture the voices of those living with the disease and those at the frontline of service delivery, to share learned experiences and practices for learning and change.

The “Rethinking Malaria in the Context of COVID-19” global engagement included Key Informant Interviews, group, and one-on-one meetings with stakeholders, including frontline health providers, malaria experts, other public health experts, and health researchers. Meetings with external advisory committee members across workstreams to review initial findings were also conducted. Over 200 individuals primarily from Africa were engaged over a multi-month long period (39% female and 61% male) to offer perspectives from academia/ research institutions, government, multilateral organizations, civil society, and the business sector.

Notes from the discussions were converted into a matrix to draw out key themes; six key themes were identified as bridging comments across respondents. These themes highlight high-level findings on how to integrate malaria services for improving health outcomes, particularly in high-burden countries. This paper presents a summary of findings and recommendations within each theme. As a next step, it will be critical to translate these results and recommendations to diverse audiences by crafting unique messages for different constituencies depending on their needs and positions [18].

### Key theme #1: It is important for the malaria community to contribute toward building strong, resilient, and sustainable health systems

A strong and resilient health system is a critical foundation for integrated service delivery that is sustainable [19]. While some vertical services—such as long-lasting insecticidal net (LLIN) campaigns—may be delivered efficiently outside the health systems [20], strong, resilient, and responsive primary health care systems are necessary to make progress against malaria [17] and to ensure that countries can better respond to threats like emergencies and pandemics [21]. Strong primary health care becomes even more critical as the malaria burden declines to identify and manage these cases in the “last mile” of the journey toward “zero malaria” [22–24].

We conceptualize the health system as including other sectors critical to its functioning, including agriculture, the environment, housing, roads, water, sanitation, and education, among others. For instance, roads are critical in access to health services, including diagnosis and treatment of malaria [25]; poor sanitation increases malaria burden [26, 27], and well-planned urbanization can help to reduce malaria transmission through the destruction of mosquito breeding sites and improved housing [28, 29]. To achieve the shared objective of optimum health for the population, the malaria community should work closely with stakeholders across sectors to strengthen health systems by leveraging local networks and identifying common areas of interest and shared objectives. Countries that have eliminated malaria have worked with other ministries, departments, and agencies in the country [30–32].

The malaria community must see health system strengthening as core to its mission. Integration also needs to reach all parts of the health system, including currently “disease siloed” activities like financing of programme activities, supply chain of programme commodities, training and supervision of staff, and some persisting parallel health management information systems (HMIS) for malaria [17]. Some prevention interventions such as ITN (mass campaigns), indoor residual spraying (IRS), larviciding, and mass drug administration (MDA) are admittedly challenging to integrate. In contrast, others, such as continuous distribution of ITN and intermittent preventive treatment for malaria among pregnant women (IPTp), have been integrated into the maternal and child health service delivery [33–35].

Despite its importance, there is an insufficient emphasis on actual health system strengthening within malaria and other disease control efforts [36]. Some of this is due to donor and target-driven activities that aim at system level short to medium-term effects. Health system strengthening focuses on comprehensive changes to performance drivers, such as policies and regulations, organizational structures, and relationships across the health system to motivate changes in behavior, allowing more effective use of resources to improve multiple health services as against health system support which improves outcomes primarily by increasing inputs [36]. Therefore, actual health system strengthening interventions are far-reaching and sustainable, achieving system-level effects instead of impact at the organizational level.

An analysis of funding requests carried out by the Technical Review Panel of the Global Fund to Fight HIV/AIDS, Tuberculosis, and Malaria (GFATM) that included investments in Resilient and Sustainable Systems for Health (RSSH) submitted in the 2017–2019 allocation period found that most (66%) proposed RSSH investments would provide support to—rather than strengthen—the health system [37]. Similarly, an evaluation commissioned by the Technical Evaluation Reference Group of the GFATM that examined funding requests from 11 countries over the same period also showed that only 27% of RSSH investments from the main allocation funding request were used for health systems strengthening [38]; most of the funding meant for health systems strengthening was directed towards activities that supported rather than strengthened the health system.

The malaria community must expand beyond the “malaria box” and cede some control to other stakeholders. Potential partners—such as the faith/religious community, academia, the military, and the private sector—could make critical contributions to malaria elimination efforts as they did for COVID-19 [39–41]. The malaria community will need to “speak the language” familiar to others to engage these partners in potentially meaningful action (see Key Theme 4). The mindset of malaria program implementers must change from seeing clients as “belonging” to a specific program to building a holistic and resilient health system in which people with any disease (existing disease, including malaria, or novel diseases such as COVID-19) can obtain care.

The effort to strengthen health systems should also include community health systems as a critical conduit to reach the unreached. It is important to work closely with and listen to community members to ensure co-creation and ownership of effective strategies to improve malaria outcomes. Considering the specificities of each intervention and context is paramount for the effectiveness of delivery, and this can only be done by engaging the community and working closely with them. Sri Lanka eliminated malaria, and key elements included enhanced awareness and community engagement programmes [30]. The COVID-19 pandemic has also illuminated how offering services closer to communities can help reduce density at overcrowded health facilities and increase access during emergencies; the resilience of community-based ITN distribution during the COVID-19 pandemic illustrated this [42, 43]. The pandemic emphasized the importance of engaging communities as partners in managing both existing and new health challenges. Community engagement must include strategies to generate demand for services and broader community support for programmes. These include linking services or integrating them with existing ones that are highly valued and involve community gatekeepers. There are lessons from malaria and other areas, such as in sexual and reproductive health fields [44, 45]. This further reinforces the importance of building and fostering the robust implementation of malaria control—and other public health—interventions at the community level.

### Key theme #2: Reframe malaria as an equity issue

The burden of malaria is not equally borne by all members of society [46]. Not all countries, communities, or individuals start at the same place—history, culture, politics, economics, social communities and other contextual factors all work hand-in-hand in ways that affect equity and malaria outcomes [47–53].

We encourage thoughtful deliberation about what “equity” means within the malaria community. How will we know when equity has been achieved? Are we seeking equity in access to technologies and commodities, services, or equity in outcomes such as incidence or mortality? Trade-offs and potential efficiency losses within countries (e.g., across groups or subnational regions) or across countries (e.g., at regional and global levels) may complicate these decisions, so they should be carefully considered.

Governments of high-burden countries should consider mechanisms to reduce inequities, such as National Health Insurance schemes with arrangements that cater to the poor and vulnerable [54, 55]; and multi-level and intersectional strategies to address the social determinants of malaria burden informed by a more nuanced understanding of how social forces impact malaria burden. For example, suppose research highlights a disproportionate burden among adolescent out-of-school girls or migrant workers who live in temporary low-quality housing. In that case, the corresponding stakeholders and government ministries can be involved in relevant policymaking. Although analyses have modeled the potential impact of multisectoral action on malaria burden [56], this has not resulted in sustained involvement of broad stakeholders in the malaria response. There may be little incentive for those beyond the Ministry of Health to engage in malaria control discussions until their participation in the underlying risk factors—and consequently their essential role in the response—is made clear. Additionally, engaging more stakeholders may widen the fiscal space and introduce new perspectives for formulating and implementing strategies for integrated service delivery to control malaria [57]. (See more on engaging diverse stakeholders in Key Theme 4.)

Greater attention to heterogeneous risk and social determinants of burden also presents an opportunity to engage social movements, civil society, and civic activism in affected countries. One major area of opportunity is the powerful women’s movement. By acknowledging the essential role of women and girls in the fight against malaria—as caregivers and decision-makers in their households and as leaders in governments and societies—the malaria community could leverage a significant source of social and political strength. This applies to every aspect of malaria control—women can lead advocacy efforts for increased local investments in malaria (see Key Theme 5), but they must be appropriately equipped with information (see Key Theme 3) and financially empowered to do so. Existing limitations, such as gender inequality and economic disparities in many high-burden countries, may have hindered such efforts to date [58], so donors and other stakeholders must be more intentional in designing mechanisms and incentives to harness this opportunity. Young people are also increasingly active in advocacy and policy efforts. They should be engaged and supported to organize sustainable advocacy efforts for malaria, including building on their existing climate change efforts. Collaborating with community and youth-focused movements such as “Zero malaria starts with me” and “Draw the line against malaria” may help synergize with community partnerships in the fight against malaria.

The malaria community must prioritize research *on inequities* to inform the development of strategies to mitigate these inequities. Marginalized groups will continue to be neglected by traditional malaria services. They will continue to see a disproportionate burden until we fully understand who is most vulnerable to malaria and why in different contexts, including the intersectionality of risk across demographic, geographic, and other factors. However, the malaria community has largely under-emphasized the formal study of the inequity of malaria burden and disease management.

The malaria community should be open to the expertise of social and behavioral scientists. Funders and scientific agencies like the National Institutes of Health (NIH) should finance research on the social determinants of malaria burden (i.e., incidence, morbidity, and mortality) and behaviors (e.g., access and adherence to prevention and treatment). Journals and editors should solicit articles on these topics and widen the pool of potential reviewers to give adequate and fair assessments of these papers.

### Key theme #3: Make data on malaria visible, accessible, and actionable at all levels

Integrated service delivery requires high-quality data, which needs health information systems that collate timely and complete data. The Global Malaria Programme proposes moving away from “one-size-fits-all” interventions to tailored implementation depending on the local epidemiological context, geography, disease burden, and human behaviors in high-burden countries. To do this effectively, high-quality, rapidly disseminated data are necessary. Additionally, integrated service delivery is a “data-hungry” endeavor. For example, building and running a responsive supply chain for integrated services requires detailed, local data about the occurrence and co-occurrence of health conditions. Suppose the data are not valid and reliable, resulting in misclassification of ailments, or do not provide timely information, resulting in missed seasonal or other fluctuations. In that case, supply chains will not be able to deliver appropriate commodities to the right places, for the right needs, at the right time. This will result in product wastage, stockouts, and possible mismanagement of diseases, impacting health outcomes. Therefore, data systems must be strengthened to enable integrated service delivery for malaria.

Existing data systems have significant gaps: private sector information may not be included because of data alignment and coordination challenges, and data on malaria interventions, especially community-based diagnosis, treatment, and prevention, also may not be consistently incorporated. These omissions mean that incomplete data are being used for decision-making. Ministries of Health and National Malaria Control Programmes must develop mechanisms for linking health information data from diverse sources and across different levels of the health system. This is essential for service integration, as a complete data picture is necessary to ensure seamless and holistic service delivery across contexts, sectors, and service delivery outlets. Routine Health Information systems also need to be expanded to include all aspects of health information related to malaria control activities that are currently included. Some essential malaria control tools—particularly those related to vector control like mass distribution of ITNs, IRS, and seasonal malaria chemoprevention—are not routinely delivered by health providers through routine health care services in all countries. Thus, measuring the implementation, coverage, and outcomes resulting from these critical interventions is not gathered as part of standard health information processes; and we must look to build data and monitoring systems that include all information from efforts to reduce the malaria burden.

Therefore, the data on malaria is not always visible to those who generate them or those represented in the data. Program implementers and frontline managers often collect epidemiologic data on malaria but do not analyze the data themselves or use the data to inform local decisions. At present, data on malaria are often generated at health facilities and included in the DHIS2, or sometimes in a stand-alone malaria information system from where they may be forwarded to regional and national levels. While much progress has been made with the introduction of the online DHIS2 platform regarding integrating data gathering and management at the district and other levels of the health system, challenges remain. Many users are still focused on data reporting, with less attention given to data analysis and use for decision-making even though analytic and visualizing functions such as dashboards have been included in the DHIS2 platform [59–62]. Furthermore, the data in the DHIS2 are available only to a few health staff who have been given distinct levels of access—some to enter health transaction data only and others to analyze the data for decision-making and reporting. However, health staff with the latter level of access are very few in any health institution. Most frontline health workers, therefore, may not have complete visibility of the data they contribute to, even at their level, and are therefore unable to appreciate where they have come from, where they are, and where they are going regarding their efforts in the control and elimination of malaria.

We need a shift in mindset: from one where data satisfies the needs of others at a higher level to one where there is ownership and need for data at the level where the data are generated. Every stakeholder involved in the control of malaria, whether in service delivery, research, or policymaking, has a role to play in gathering, analyzing, and using information. Those who tend only to gather data must be equipped to analyze/interpret and use it at the level where it is collected. Efficient and effective use of data for decision-making, planning, and implementation should be incentivized to catalyze this shift in mindset. There is also a need for investment in technological and other innovations to make malaria data more succinct, available, and accessible at all levels.

Data must be made available in a format that is understandable, user-friendly, and accessible in a timely manner, particularly at the level where they are generated. Several malaria dashboards have been developed for use in various settings. Still, these may not be in a format accessible to or useable by all potential users, including at the community level.

**Fig 3:**
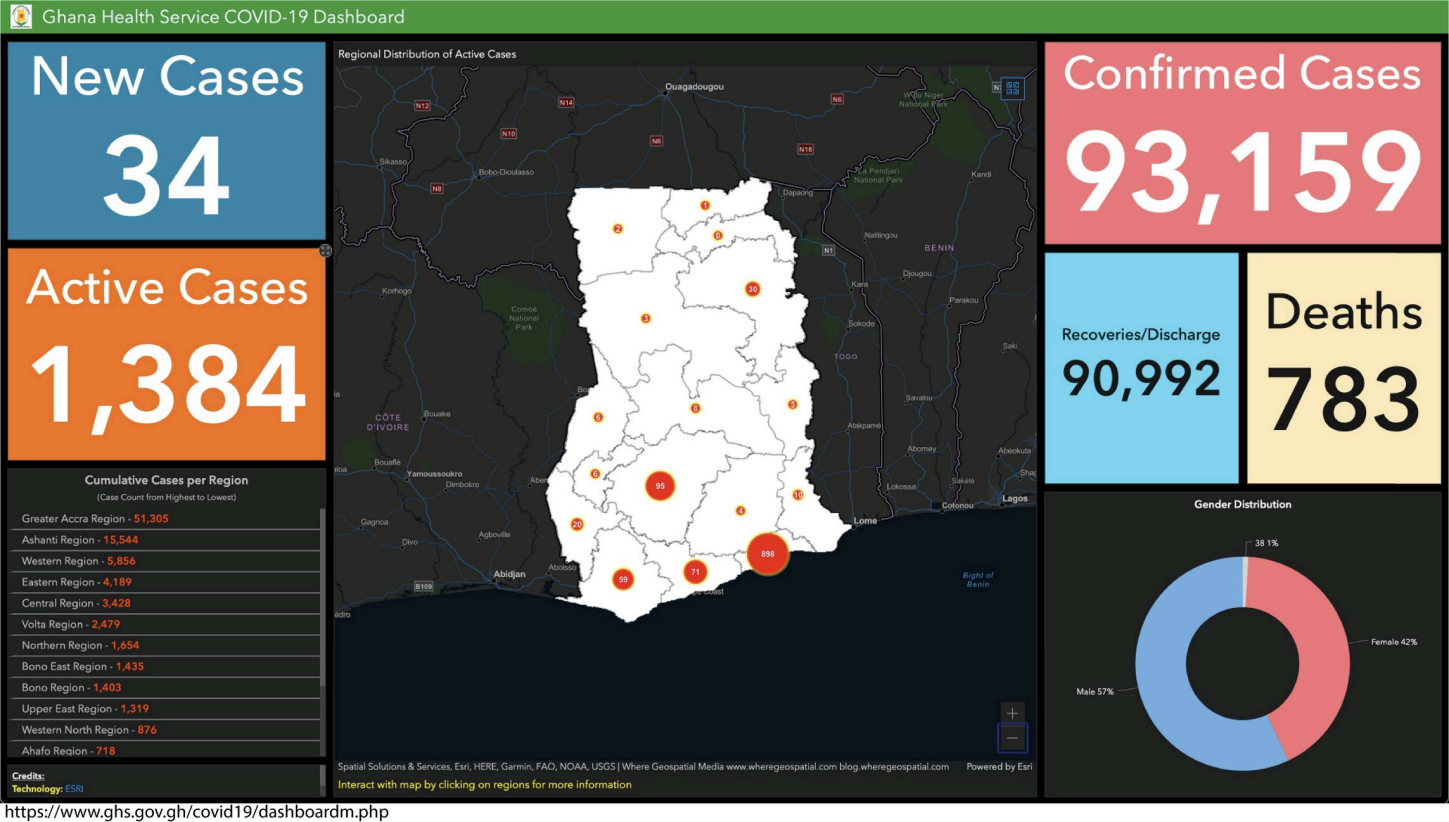
Sample malaria dashboard based on COVID–19 dashboard data points.

Data on COVID-19 offer a lesson for the malaria community. Countries and sub-national settings regularly report a few key indicators; these are updated rapidly to allow comparison within and between countries. The malaria community likewise needs to invest in better data collection platforms and systems and mechanisms to disseminate information in a simple form that can be appreciated by diverse groups, from citizens to health workers and those at various levels of government. Reported data elements might include the number of confirmed malaria cases, number of malaria deaths, number of malaria-related hospital admissions, coverage of preventive methods (e.g., bed nets, intermittent preventive treatment among pregnant women), and cost per malaria episode. An example proposed malaria “dashboard,” modeled off the Ghanaian Ministry of Health’s COVID-19 dashboard, is shown in Fig 3.

Likewise, citizens need to be informed and empowered to hold their leaders accountable. Accountability can help ensure that malaria programs and policies are responsive to community needs—and robust and accessible data are central to increasing accountability. High-quality, disaggregated data can help those who plan and deliver programs to design stronger, locally tailored strategies. Citizens can then use these data to make informed assessments of their level of satisfaction with these decisions made. This cycle of data-driven accountability itself creates incentives for generating more and better-quality data. Lessons on holding mayors and other elected leaders accountable, working collaboratively with local stakeholders, and using current health data can be learned from the Fast-Track Cities strategy in HIV, which involves over 380 cities and their elected leaders [63]. Approaches like performance-based financing and expanded use of community scorecards—wherein communities and health providers agree on indicators and regularly and collaboratively monitor them—have been proposed as strategies to achieve local accountability [64–66]. However, effectively implementing these requires investments in increasing capacity for data-driven decision-making and data democratization to allow people of all backgrounds to understand the malaria situation.

As the local situation changes, leaders must also be skilled and empowered to use data for decision-making and course-correct based on data. Capacity requirements for public health leadership positions should be broadened beyond core technical skills to include data fluency and "soft skills" such as working with diverse stakeholders and managing their interests for the common good. National Malaria Control Programmes can take advantage of recent technological advancements to achieve timely dissemination of accurate data to diverse audiences. This can include moving beyond traditional media (e.g., television and radio spots), which may not be a frequent or trusted source of information, especially by youth. Nearly half of sub-Saharan Africans own a mobile phone [67], which offers a powerful social and behavioral change tool [68]. Additionally, using a credible source to disseminate locally relevant and high-quality data may help combat misinformation, myths, and rumors.

High-quality malaria data systems will benefit other disease areas as well. Existing surveillance networks were instrumental during COVID-19 in many high-malaria burden countries; malaria data scientists have expertise in and procedures for diagnosing, reporting and tracking febrile cases, which presented clear opportunities during the early phases of the COVID-19 pandemic. If malaria data systems report data in a reliable and timely manner, this could help detect new fever hotspots and catch disease outbreaks early. Similarly, COVID-19-related investments in surveillance and data system infrastructure could be leveraged for the malaria community if (or when) the pandemic’s “acute” stage recedes.

### Key theme #4: Make a case for investing in malaria that resonates with diverse stakeholders

The global malaria community must make a compelling case for malaria that advocates for integrated services and frames this as an investment rather than an expense. Although stakeholders in the public health space may be motivated by health-related outcomes and objectives, this may not resonate with the broad coalition of policymakers, activists, politicians, and individuals who should be engaged more actively in the malaria response (see Key Themes 2 and 3). This coalition includes both global and domestic stakeholders. With increased service integration and ultimately the move toward UHC, this coalition will diversify even further, and the investment case will need to adjust accordingly.

First, we urge rethinking the malaria community’s conceptualization of “elimination.” For countries far from this goal, its ambitiousness risks discouraging policymakers and practitioners. For people living in high-burden countries where malaria is ever-present, there is a disconnect between their lived everyday experience and this objective. Elimination of malaria must be seen as a continuum with communities, districts, and countries along the continuum of malaria transmission. From global agencies to national and subnational entities, we propose that policymakers reconsider the framing of malaria goals and identify nearer-term objectives. These should form the basis for malaria plans and policies, with realistic timeframes. Incremental gains on the path to elimination should also be acknowledged and celebrated. Realistically, there is no short-term path to malaria eradication for high-burden countries, so the language and commitments of donors and policymakers should instead move toward a long-term trajectory. This requires corresponding shifts in how malaria financing is conceptualized and offered; as countries progress through incremental improvements, the mosaic of funding sources, policies, clinical guidelines, and programs also must evolve. Expectations about reasonable outcomes, timeframes, and costs should adjust as countries progress on this trajectory. While it is essential to leave no one behind, we also must recognize that there will be varied progress across and within countries. Program implementers, policymakers, frontline managers, and affected communities must jointly own and celebrate successes along the way as they focus on “control” while considering the longer-term objective of “elimination.”

Second, although malaria causes a substantial disease burden, it may not be viewed as a high-priority issue even among those most affected. National governments must align their priorities—and spending—with local needs and priorities. Identifying a compelling and meaningful goal is therefore essential. Other conditions, such as living necessities (e.g., housing, food, employment) and other health issues, may be more top-of-mind [69]. Improved data collection and reporting of malaria statistics in a meaningful and “provocative” way (see Key Theme 3) may shift some people’s perspectives. Still, the malaria community should be aware that other health indicators may carry more salience. Other disease areas have similarly worked to make compelling investment cases that leverage other indicators (including health-, social- and economic-related), such as HIV and maternal health communities—and these examples may be instructive for malaria advocates. A framework that considers overall health and wellbeing—to which malaria contributes only one aspect—may be more compelling to some. This may be particularly true in the context of UHC and PHC and the increasing emphasis on building resilient and comprehensive health systems that address health as a multidimensional construct. However, we must note that such broader goals may be less compelling for donors and international agencies that rely on vertical programs’ tightly prescribed measurements for monitoring and evaluation. This recommendation is thus tied closely to the key concepts of service integration and the need for a fundamental rethinking of what malaria progress means in the context of a world moving toward UHC.

Third, the malaria community must commit to developing and disseminating investment cases that “speak to” diverse audiences. Although there have been numerous analyses of the cost-benefit of investing in malaria [3], these may not reach the desks of influential stakeholders outside the public health community (e.g., policymakers in other ministries such as finance). This will require a candid reconsideration of who is motivated by what information and the most effective conduits for these messages. It also may be necessary to expand our evidence base to build compelling investment cases: for example, conducting local or regional analyses that emphasize the benefits of in-country or in-region manufacturing of commodities or the context-specific particularities of program implementation. Additionally, in light of the recommendation above to reconsider endpoints, investment cases should be constructed to allow flexibility in outcomes that are most compelling and salient to different stakeholders and at other points in time. This might include economic indicators such as productivity or jobs creation and sustainment, alongside health indicators. This would be a substantial undertaking, so donors and research funders should support such research and knowledge translation efforts. Reframing malaria as a compelling investment rather than an expenditure is a necessary step toward building national and local buy-in for malaria control efforts.

Lastly, we highlight a central recommendation throughout this theme: the importance of elevating and amplifying local voices. The malaria response must not continue to be “driven” by donors and so-called experts from high-income countries. On everything from R&D to implementation to measurement, local expertise must be moved to the forefront. The current investment case for malaria—what to do, why to do it, how to do it—is primarily driven by global agencies and technical experts from high-income countries. This should change. Every health program should work toward the priorities of those in the most-affected regions, not in response to the goals and mission of the donor agency. This will require international agencies, donors, and scientists to reposition themselves as active allies to researchers, policymakers, communities, and organizations in affected countries. There must be an investment in, and commitment to, local and regional responses to malaria.

### Key theme #5: Improve access to current information on best practices in the implementation and integration of malaria interventions

An effective drug, diagnostic, vaccine, or prevention technology is necessary but insufficient to achieve improved health outcomes. Critically, implementation science provides the theories, frameworks, and methods to help plan, design, deploy, adapt, and evaluate strategies to increase the impact of proven-efficacious technologies and interventions [70]. Implementation scholarship emphasizes the measurement of implementation outcomes (e.g., acceptability (or feasibility), fidelity, costs, sustainability) [71]. This orientation toward the “how” of implementation could transform the malaria community. It would comprise a more conscientious, systematic approach to implementation for Ministries of Health and partner organizations, with a corresponding broadened set of monitoring indicators and new opportunities for knowledge generation and translation into updated policies, clinical guidelines, and programmatic, technical guidance.

The Global Malaria Programme recently launched an easily accessible online platform (called MAGICapp), aiming to provide a “one-stop-shop” for guidelines and recommendations with supporting evidence for use by programme implementers at all levels. While this is an excellent initiative, it must be accompanied by equipping implementers with additional skills to adapt guidelines and recommendations to diverse contexts and subsequently implement, scale-up, and sustain interventions.

**Fig 4:**
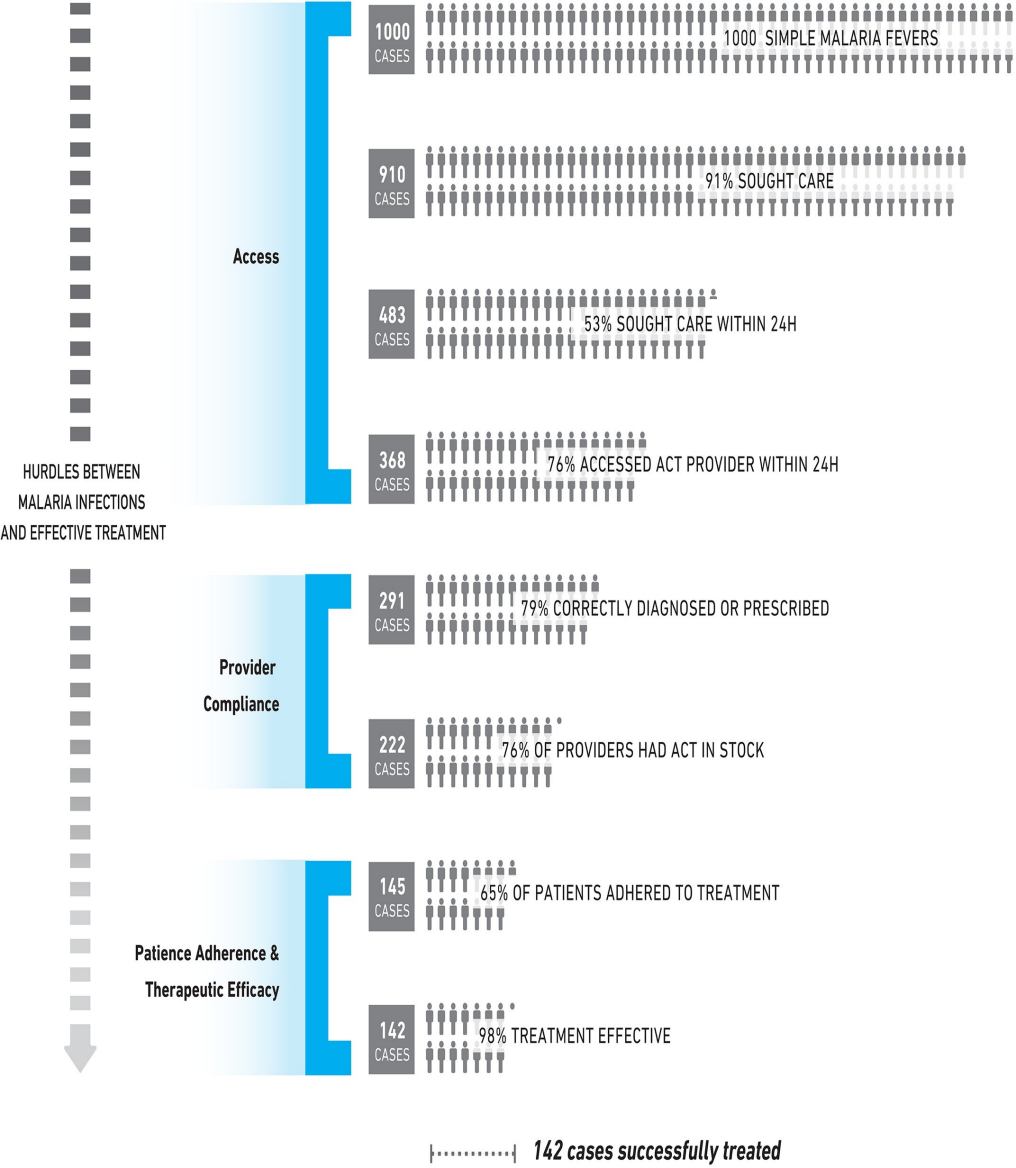
Efficacious interventions lose traction as they “travel” through health systems (Adapted from WHO/TDR implementation research toolkit).

We need to contextualize implementation, understanding that real life differs from experimental situations. The malaria community has traditionally assumed that proven-efficacious tools should work equally well in all settings. Yet studies repeatedly demonstrate that efficacious interventions can lose traction as they are implemented in the health system, resulting in much lower effectiveness (Fig 4). This is seen clearly when highly efficacious vector control tools are distributed in one place and do not seem to work as well as they had worked elsewhere [72]. To increase the effectiveness of interventions in the “real world,” implementers and other stakeholders need to identify implementation challenges and design strategies to address them.

Research in this area must be further encouraged. It aligns with the Global Malaria Programme’s shift from a “one-size-fits-all” approach to implementation to a process that considers the specific context and its stakeholders and works with them to optimize performance. It also aligns with the shift from viewing countries in distinct categories of “control,” “consolidation,” “pre-elimination,” and “elimination” to recognizing countries along a continuum towards elimination that requires iterative planning with anticipation of transition and evolving approaches at national and sub-national levels [73].

While malaria program implementers are well-equipped with core technical skills, implementation science skills are critically needed to improve the effectiveness of malaria interventions in various settings. Implementers must be equipped to consider the social and behavioral science that drives implementation and not to expect interventions to work similarly and achieve the same results everywhere; and knowledgeable in the foundational concepts of implementation science, which will enable them to identify where specific target beneficiaries of a particular intervention are on the diffusion–dissemination–implementation–adoption–sustainability continuum, at any point in time [74]. This will ensure flexibility and responsiveness to changing contexts as the malaria burden evolves and novel disease and implementation challenges arise.

### Key theme #6: Facilitate research and innovation to develop new solutions in and by the most affected regions and countries

The importance of research and innovation in accelerating progress toward the elimination of malaria cannot be overemphasized. New tools and strategies—developed through research and innovation—are needed to catalyze progress and are fundamentally linked to the themes around integrated service delivery. They will have limited impact without improved service delivery and integration. There is thus a deep interconnectedness between R&D and service integration.

Local research institutes in high-burden settings must promote research and innovation, particularly around tools to facilitate service integration. For example, several diseases present akin to malaria and can only be differentiated using diagnostic tools. The COVID-19 pandemic posed a diagnostic dilemma in outpatient departments in malaria-endemic areas. Patients presenting with fever might have malaria, COVID-19, both, or a different cause of fever altogether, such as urinary tract infection or pneumonia. We, therefore, need a point-of-care test that can differentiate between malaria and other common causes of fever, including those caused by viruses such as COVID-19. Such a tool would ensure accurate diagnoses while also serving as a multiplex entry point for primary care.

The COVID-19 pandemic brought into sharp focus how high-priority issues with significant political dedication and financial resources can massively accelerate R&D achievements. High-burden countries should lead advocacy efforts to keep malaria high on the global health agenda. R&D efforts should be a top priority for intergovernmental organizations such as the African Union and Asia-Pacific regional organizations. To achieve this, nations need to be more involved in funding themselves. Given gaps in infrastructure, technology platforms, and a lack of a critical mass of skilled scientists, there is a need to find sustainable solutions that embed local capacity development in any R&D strategy.

Additionally, the speed of COVID-19 vaccine development offers lessons for the malaria community: decision-makers must reverse the traditional, linear approach to innovation for malaria. But challenging the status quo requires unconventional methods. For example, freedom of thought in R&D may be hindered by financiers’ prescription of how to spend funds; much of the current funding comes from northern funders primarily to northern academic or other research institutions that link up with institutions in endemic countries. This creates a situation where the agenda is set not by endemic countries but by those who hold the purse [75]. It is time for endemic countries to contribute to finding solutions for what affects them most. Entrepreneurs from affected countries should be encouraged and incentivized to be more involved in developing new tools and supporting efforts to eliminate malaria from their region.

Research institutes in regions and countries that bear the greatest burden of malaria must take the lead in prioritizing projects that will provide solutions to their problems and should seek to overcome barriers, from the laboratory to the legislature, that limit progress. More local institutions in Africa and high-burden settings should engage in R&D and clinical trials. The solutions needed in the field must drive the agenda for research, development, and innovation. Dynamics in R&D partnerships, including those across other diseases, should be re-assessed to ensure that projects are prioritized not just on who holds the purse but on what is relevant for accelerating progress toward malaria elimination. Relaxation of import/export regulations can ease the acquisition and sharing of reagents and research products, facilitating priority access to developed products, including vaccines, diagnostics, drugs, and LLINs. Young researchers must be encouraged and supported to “think outside the box” for innovation.

There is also a need to establish R&D networks with strong regulatory linkages in the regions most affected by malaria, which will facilitate swift prioritization of new interventions from early-stage laboratory and clinical evaluation to large-scale implementation.

We call for papers to provide direction for re-thinking malaria in crucial R&D areas, including vector control, new and more effective drugs, vaccine development, diagnostics, and data science.

## Discussion

The objective of this working group within the “Rethinking Malaria in the Context of COVID-19” global engagement was to consider what it would mean and what it would take to achieve integrated service delivery for malaria. Through many structured discussions with stakeholders—including, critically, voices from those “at the front lines” of the malaria response in highly-affected countries—we aimed to identify challenges and corresponding opportunities. We identified six key themes, articulating specific recommendations aligned with each (Table 1). We note that this research took an inductive approach, so these themes emerged from the interviews; future research in this area should seek to identify and leverage frameworks and theories to inform deductive studies that may have increased comparability and generalizability.

**Table 1:** Challenges and corresponding opportunities to malaria service delivery integration for malaria.

Key Themes	Specific Program & Policy Action Recommendations
Contribute towards building strong, resilient, and sustainable health systems	• Engage in multi-sectoral planning and policymaking • Build local networks and collaborations and “speak the language” familiar to other stakeholders in order to facilitate meaningful engagement and action • Work closely with communities to ensure co-creation and ownership of effective strategies
Reframe malaria as an equity issue	• Invest in research on the “social determinants” of malaria burden • Emphasize intersectionality of malaria risk through responsive, multi-sectoral approaches • Expand the malaria scientific community (e.g., journal editors, reviewers, scientific conferences) to include social and behavioral scientists • Engage social movements (i.e., youth and women) in malaria activities
Make data on malaria visible, accessible, and actionable at all levels	• Leverage recent technological advancements to link data on malaria burden and service coverage from across sources and levels of the health system • Design dashboards and data reporting formats that are meaningful to all people and actionable • Empower policy and program stakeholders from national, regional, and local levels to interpret and act upon data • Empower community members to hold their local leaders accountable for the malaria burden in their locality
Make a case for investing in malaria that resonates with diverse stakeholders	• Engage in global-local dialogues about what malaria program goals should be, with attention to other competing priorities and intra-national disparities • Match timeframes in funding and policies to what is reasonable for the goals stated • Foster research to conduct salient investment cases for malaria, with leadership of local scientists, input of local data, and selection of locally meaningful endpoints/outcomes • Invest in systems for knowledge translation, to empower advocates, community groups, and diverse coalitions of policymakers, with information necessary to recast malaria control as an investment not an expenditure
Increase research into the best practices of implementation and integration for malaria	• Prioritize dissemination and implementation science research to identify strategies for increasing the uptake and impact of malaria prevention, diagnosis, and treatment technologies • Equip implementers with implementation science skills to enable them to have the “know how” for integration of malaria with other health programs and services
Facilitate research and innovation toward the development of new solutions in and by the regions and countries that are most affected	• Local entrepreneurs from high-burden countries should be encouraged and involved in investing in developing new tools for malaria, particularly those that promote and facilitate service integration • High-burden countries should lead advocacy efforts to keep malaria high on the global health agenda. • Research groups from affected countries should drive their own local R&D efforts

Bearing this in mind, and throughout the key themes articulated above, several changes in mindset and terminology may be necessary. We urge deep thought and urgent action about de-colonizing malaria programs and policies: these should be prioritized, designed, implemented, and evaluated by the communities most affected. We encourage a de-commoditization in the malaria response; effective products are necessary, but health services and systems are the “engine” that will enable us to eliminate malaria. Emphasis must be placed on implementation, the development, and dissemination of new tools, and the malaria community needs to engage more meaningfully in ongoing broader discussions (e.g., around climate change and health systems strengthening).

Many disease-specific communities are grappling with what it will take and what it will mean to move toward UHC. We encourage the malaria community to leverage its broadly applicable knowledge and unique position in the health system to seize the opportunity to take a leadership role in strengthening health systems and moving towards integrated services and, ultimately, universal health coverage. For example, many countries’ COVID-19 pandemic responses were led by local malaria experts, as they possessed the relevant experience and skills in disease surveillance and diagnostics [76]. (Incidentally, shifting malaria staff to another disease area for nearly two years may have unintended consequences on malaria control efforts—an area worthy of rigorous study.) If the malaria community improves data systems and surveillance activities, strengthens supply chains and other health systems infrastructure, builds health worker expertise, and innovates new service delivery paradigms, these “railroad tracks” could also be used by other health programs—resulting in improved health outcomes for all. Integrated community case management programs have made headway on this, but much more remains to be done.

This is not a new idea: integration has been discussed for decades, including in the malaria community. Numerous technical, strategic documents and country strategies have emphasized integration. Despite these years of dialogue, integration for malaria control has not yet gained traction at the policy and implementation levels [77]. Why is this the case, and how can the malaria community move past this to make meaningful progress? It is time for the global community of malaria policymakers, planners, implementers, researchers, clinicians, and partners to think deeply and humbly about why there has been so little progress on integration and interpret any feelings of déjà vu with introspection rather than egoism. It is a sign of failure to endorse the same ideas for decades without making meaningful progress. Just as the global community lent weary and skeptical ears to repeated warnings of an inevitable pandemic, and was consequently under-prepared, repeated recommendations about integration in the context of malaria are not resulting in the necessary action. This must change. In the context of UHC and pandemics, it is even more critical to seriously and specifically address the integration of malaria services.

One possible reason why progress on integration has been slow is that all communities are different. There is no one conversation or “solution” that will fully resolve this for all geographies and populations. We, therefore, encourage a context-driven approach to integration. It should also be noted that evidence on integration from other diseases is mixed [78–80], and the malaria community could learn many lessons from other “vertical” programs that have attempted integration, including HIV. Integrating malaria prevention and control efforts with other services must be considered and approached cautiously, bearing the context in mind.

Lastly, we wish to explicitly acknowledge that expecting to “solve” the issue of integration through a global approach and broad discussions that are not tailored to the specific needs of different countries—nor the diverse communities within these—itself perpetuates the colonialist legacies of malaria control efforts [81]. Top-down decision-making that occurs outside the most-affected communities is not the answer. Diversity of voices and a variety of solutions are needed. But burdening the most-affected countries and communities with designing policies and programs to overcome the myriad wrongs perpetrated by the Global North—actions that set the course of malaria burden and malaria control to where it is today would be an injustice. This is the time for local leaders and communities to devise a path forward and out of the cycle of discussion and debate about integration. However, the global malaria community cannot abdicate its duty by placing the responsibility for this work on high-burden countries. Governments, donors, academics, and stakeholders from high-income countries must support and amplify these local conversations and provide the tools and resources needed in this global effort to re-think malaria. Their role should be as allies, partners, and accompaniers.

Central to the notion of “rethinking malaria” in the context of integrated service delivery is a reconsideration of where we are and where we are headed. If the global community is pointed toward Universal Health Coverage, malaria programs and policies must figure out where they fit. As outlined above, this will require transformations in all aspects and processes toward eliminating malaria. The impact of integration is likely to be substantial—but only if all the requisite building blocks are strong enough to support it.
